# Supplementary material for: Multilevel Weighted Support Vector Machine for Classification on Healthcare Data with Missing Values
Source: PLoS One. 2016 May 19;11(5):e0155119. doi: 10.1371/journal.pone.0155119 (PMC4873242; doi:10.1371/journal.pone.0155119)
Supplement: S1 File — Table A, Comparative sensitivity results for ML(W)SVM against the regular SVM, WSVM, NB, C4.5, 5NN, and LR on Twonorm, Letter, Ringnorm, and Clean academic datasets for different fractions of missing values (rmv) using the REM imputation method. Table B, Comparative specificity results for ML(W)SVM against the regular SVM, WSVM, NB, C4.5, 5NN, and LR on Twonorm, Letter, Ringnorm, and Clean academic datasets for different fractions of missing values (rmv) using the REM imputation method. Table C, Comparative accuracy results for ML(W)SVM against the regular SVM, WSVM, NB, C4.5, 5NN, and LR on Twonorm, Letter, Ringnorm, and Clean academic datasets for different fractions of missing values (rmv) using the REM imputation method. Table D, Computational time (sec.) for C4.5, 5NN, NB, LR, and MLSVM (excluding model selection) on public datasets. The results show that MLSVM is faster than other machine learning methods. In addition, we note that the average computational time of the REM imputation for public datasets over all missing value ratios are: Twonorm 1.22, Letter 6.89, Ringnorm 1.18, cod-rna 33.76, Clean 7.85, Advertisement 0.57, Nursery 1.41, Hypothyroid 0.16, Buzz 1705.60 sec. respectively. (PDF) [file pone.0155119.s001.pdf]

1 Supplementary Materials

Details about the REM method

- The parameter P and θ are calculated based on Shannon information theory [1].
- To implement this algorithm, we refer the reader to [2] in which the implementation is provided. The EM algorithm in this package is based on iterated linear regression analyses. In the regularized EM algorithm, a regularized estimation method substitutes the conditional maximum likelihood estimation of regression parameters in the conventional EM algorithm. The modules in [2] present the truncated total least squares (with fixed truncation parameter) and ridge regression with generalized cross-validation as regularized estimation methods. In our application, we used multiple ridge regression, stagnation tolerance parameter = 1e-2 (stop criteria for iteration), maximum number of EM iterations = 50, truncation parameter selection: 'KCV' (that chooses a truncation adaptively for each record by K-fold cross-validation), parameter K = 5 for K-fold cross-validation, norm of error to be minimized in K-fold cross-validation = 2.

Description of the cross validation scheme

- We performed 10-cross validation on the data. For cross validation purposes, 90% of the data was used for training and the rest 10% was used for testing. The data is normalized prior to classification, so that it has zero mean and unitary standard deviation. At each fold, first the training set is coarsened level by level until we reach the coarsest level and then the parameter optimization (UD) is applied to find the optimal C, C^+, C^-, and γ. Next, we train (W)SVM and get the support vectors. Then, we update the training data based on the support vectors (SVs) of the coarse level with points in the fine level that are close to SVs. Finally, we apply the UD on the updated training data with setting of optimal C, C^+, C^- and γ of previous level as corresponding initial paramenters for the next finer level training. We will continue till we reach the finest level. At the finest level we calculate the performance measure for that fold and continue to run the same V-cycle model (MLWSVM) ten times for each training and testing set that are selected randomly each time to make sure that all parts of the data are considered. Finally, we report the average performance measures over 10-fold cross validation.

MLWSVM framework

- The source code will be available at https://people.cs.clemson.edu/ isafro/software.html after final acceptance of the paper.

Supporting tables

- Tables A,B,C show the sensitivity, specificity, and accuracy of comparative algorithms on public data sets.
- Table D shows the computational time for C4.5, 5NN, NB, LR, and MLSVM (excluding model selection) on public datasets.

Table A. Comparative sensitivity results for ML(W)SVM against the regular SVM, WSVM, NB, C4.5, 5NN, and LR on Twonorm, Letter, Ringnorm, and Clean academic datasets for different fractions of missing values (r_{mv}) using the REM imputation method.

Dataset	r_{mv}	MLSVM	MLWSVM	SVM	WSVM	C4.5	5NN	NB	LR
Twonorm	5%	0.97	0.98	0.97	0.98	0.87	0.97	0.97	0.97
	10%	0.98	0.98	0.97	0.97	0.85	0.96	0.97	0.97
	20%	0.99	0.99	0.99	0.99	0.86	0.97	0.98	0.98
	40%	0.96	0.97	0.97	0.97	0.88	0.97	0.96	0.97
Letter	5%	1.00	1.00	1.00	1.00	1.00	0.99	0.96	0.97
	10%	1.00	1.00	1.00	1.00	1.00	1.00	0.97	1.00
	20%	0.99	0.99	0.99	0.99	1.00	1.00	0.97	1.00
	40%	1.00	1.00	1.00	1.00	1.00	1.00	0.97	0.99
Ringnorm	5%	0.97	0.98	0.97	0.98	0.90	1.00	0.99	0.81
	10%	0.96	0.96	0.96 S	0.96	0.91	1.00	0.99	0.81
	20%	0.98	0.98	0.98	0.98	0.91	1.00	0.99	0.81
	40%	0.96	0.98	0.98	0.98	0.90	1.00	0.99	0.81
Clean	5%	1.00	0.99	0.99	0.99	0.89	0.99	0.86	0.99
	10%	0.99	0.99	0.99	1.00	0.86	0.99	0.86	0.98
	20%	1.00	1.00	1.00	1.00	0.80	1.00	0.90	0.99
	40%	1.00	1.00	1.00	1.00	0.95	0.99	0.87	0.98
Forset	5%	0.98	0.97	0.99	0.99	1.00	1.00	0.99	1.00
	10%	0.96	0.98	0.97	0.98	1.00	1.00	0.99	1.00
	20%	0.98	0.97	0.96	0.97	1.00	1.00	0.99	1.00
	40%	0.98	0.96	0.97	0.97	1.00	1.00	0.99	1.00

References

1. Li H, Zhang K, Jiang T. The regularized EM algorithm. In: Proceedings of the national conference on artificial intelligence. vol. 20. Menlo Park, CA; Cambridge, MA; London; AAAI Press; MIT Press; 1999; 2005. p. 807.

2. Schneider T. Analysis of incomplete climate data: Estimation of mean values and covariance matrices and imputation of missing values. Journal of Climate. 2001;14(5):853–871.

Table B. Comparative specificity results for ML(W)SVM against the regular SVM, WSVM, NB, C4.5, 5NN, and LR on Twonorm, Letter, Ringnorm, and Clean academic datasets for different fractions of missing values (r_{mv}) using the REM imputation method.

Dataset	r_{mv}	MLSVM	MLWSVM	SVM	WSVM	C4.5	5NN	NB	LR
Twonorm	5%	0.98	0.97	0.98	0.97	0.87	0.97	0.98	0.98
	10%	0.99	0.98	0.97	0.97	0.86	0.97	0.98	0.97
	20%	0.97	0.97	0.97	0.97	0.87	0.97	0.98	0.98
	40%	0.98	0.99	0.98	0.99	0.88	0.97	0.98	0.98
Letter	5%	0.95	0.99	0.98	0.98	0.95	0.98	0.77	0.68
	10%	0.96	0.99	0.97	0.98	0.97	0.95	0.77	0.65
	20%	1.00	1.00	1.00	1.00	0.94	0.96	0.80	0.64
	40%	0.90	0.94	0.91	0.94	0.95	0.97	0.79	0.69
Ringnorm	5%	0.98	0.99	0.98	0.99	0.91	0.38	0.98	0.72
	10%	1.00	1.00	1.00	1.00	0.91	0.38	0.98	0.72
	20%	0.98	0.99	0.98	0.99	0.91	0.37	0.98	0.71
	40%	1.00	0.99	0.98	0.99	0.90	0.37	0.98	0.71
Clean	5%	0.99	0.99	0.98	1.00	0.78	0.85	0.74	0.80
	10%	0.98	1.00	0.99	1.00	0.81	0.84	0.73	0.81
	20%	0.98	0.99	0.99	1.00	0.86	0.82	0.70	0.80
	40%	1.00	1.00	0.99	1.00	0.71	0.85	0.72	0.81
Forset	5%	0.84	0.84	0.83	0.84	0.82	0.76	0.65	0.00
	10%	0.87	0.87	0.86	0.87	0.78	0.73	0.61	0.00
	20%	0.85	0.87	0.84	0.85	0.79	0.71	0.60	0.00
	40%	0.79	0.84	0.80	0.83	0.73	0.67	0.54	0.00

Table C. Comparative accuracy results for ML(W)SVM against the regular SVM, WSVM, NB, C4.5, 5NN, and LR on Twonorm, Letter, Ringnorm, and Clean academic datasets for different fractions of missing values (r_{mv}) using the REM imputation method.

Dataset	r_{mv}	MLSVM	MLWSVM	SVM	WSVM	C4.5	5NN	NB	LR
Twonorm	5%	0.98	0.98	0.98	0.98	0.79	0.97	0.98	0.98
	10%	0.98	0.98	0.98	0.98	0.78	0.97	0.97	0.97
	20%	0.99	0.99	0.99	0.99	0.78	0.97	0.98	0.98
	40%	0.97	0.97	0.97	0.97	0.78	0.96	0.97	0.97
Letter	5%	0.99	1.00	0.99	1.00	0.99	1.00	0.97	0.98
	10%	0.99	1.00	0.99	0.99	0.99	1.00	0.96	0.99
	20%	1.00	1.00	1.00	1.00	0.99	1.00	0.96	0.98
	40%	1.00	1.00	1.00	1.00	0.99	1.00	0.96	0.98
Ringnorm	5%	0.98	0.98	0.98	0.98	0.85	0.69	0.99	0.76
	10%	0.98	0.98	0.98	0.98	0.84	0.69	0.99	0.76
	20%	0.98	0.98	0.98	0.98	0.85	0.69	0.98	0.76
	40%	0.98	0.99	0.98	0.99	0.84	0.69	0.98	0.76
Clean	5%	1.00	1.00	1.00	1.00	0.86	0.96	0.84	0.95
	10%	0.99	0.99	0.99	0.99	0.85	0.98	0.85	0.95
	20%	1.00	1.00	1.00	1.00	0.79	0.97	0.87	0.96
	40%	1.00	1.00	1.00	1.00	0.91	0.97	0.85	0.95
Forest	5%	0.97	0.98	0.99	0.99	0.99	0.99	0.98	0.98
	10%	0.96	0.98	0.98	0.98	0.99	0.99	0.98	0.98
	20%	0.98	0.96	0.97	0.97	0.99	0.99	0.98	0.98
	40%	0.97	0.95	0.98	0.96	0.99	0.99	0.98	0.98

Table D. Computational Time (sec.) for C4.5, 5NN, NB, LR, and MLSVM (excluding model selection) on public datasets. The results show that MLSVM is faster than other machine learning methods. In addition, we note that the average computational time of the REM imputation for public datasets over all missing value ratios are: Twonorm 1.22, Letter 6.89, Ringnorm 1.18, cod-rna 33.76, Clean 7.85, Advertisement 0.57, Nursery 1.41, Hypothyroid 0.16, Buzz 1705.60 sec. respectively.

	C4.5	5NN	NB	LR	MLSVM
Twonorm	0.79	0.71	0.46	0.47	**0.35**
Letter	0.31	0.69	0.05	0.06	**0.05**
Ringnorm	1.29	1.11	0.91	0.90	**0.58**
cod-rna	9.88	9.32	9.04	9.07	**7.87**
Clean	4.71	4.01	3.38	3.78	**3.00**
Advertisement	10.25	10.05	16.04	19.91	**9.72**
Nursary	0.47	0.77	0.78	0.89	**0.40**
Hypothyroid	0.18	0.29	0.22	0.26	**0.15**
Buzz	486.04	580.62	468.17	479.94	**419.92**
